# Genetic characterization of a *Sorghum bicolor* multiparent mapping population emphasizing carbon-partitioning dynamics

**DOI:** 10.1093/g3journal/jkab060

**Published:** 2021-03-03

**Authors:** J Lucas Boatwright, Zachary W Brenton, Richard E Boyles, Sirjan Sapkota, Matthew T Myers, Kathleen E Jordan, Savanah M Dale, Nadia Shakoor, Elizabeth A Cooper, Geoffrey P Morris, Stephen Kresovich

**Affiliations:** 1 Advanced Plant Technology, Clemson University, Clemson, SC 29634, USA; 2 Department of Plant and Environmental Sciences, Clemson University, Clemson, SC 29634, USA; 3 Carolina Seed Systems, Darlington, SC 29532, USA; 4 Donald Danforth Plant Science Center, St. Louis, MI 63132, USA; 5 Department of Bioinformatics and Genomics, University of North Carolina, Charlotte, NC 27705, USA; 6 Department of Agronomy, Kansas State University, Manhattan, KS 66506, USA

**Keywords:** nested association mapping, pericarp color, genome-wide association study, genotype-by-sequencing, carbon-partitioning, multiparental populations, MPP

## Abstract

*Sorghum bicolor*, a photosynthetically efficient *C*_4_ grass, represents an important source of grain, forage, fermentable sugars, and cellulosic fibers that can be utilized in myriad applications ranging from bioenergy to bioindustrial feedstocks. Sorghum’s efficient fixation of carbon per unit time per unit area per unit input has led to its classification as a preferred biomass crop highlighted by its designation as an advanced biofuel by the U.S. Department of Energy. Due to its extensive genetic diversity and worldwide colonization, sorghum has considerable diversity for a range of phenotypes influencing productivity, composition, and sink/source dynamics. To dissect the genetic basis of these key traits, we present a sorghum carbon-partitioning nested association mapping (NAM) population generated by crossing 11 diverse founder lines with Grassl as the single recurrent female. By exploiting existing variation among cellulosic, forage, sweet, and grain sorghum carbon partitioning regimes, the sorghum carbon-partitioning NAM population will allow the identification of important biomass-associated traits, elucidate the genetic architecture underlying carbon partitioning and improve our understanding of the genetic determinants affecting unique phenotypes within Poaceae. We contrast this NAM population with an existing grain population generated using Tx430 as the recurrent female. Genotypic data are assessed for quality by examining variant density, nucleotide diversity, linkage decay, and are validated using pericarp and testa phenotypes to map known genes affecting these phenotypes. We release the 11-family NAM population along with corresponding genomic data for use in genetic, genomic, and agronomic studies with a focus on carbon-partitioning regimes.

## Introduction

Current plant resources leveraged for rapid carbon accumulation often target *C*_4_ grasses due to their highly efficient photosynthetic pathways that effectively assimilate carbon (Carpita and McCann 2008). The *C*_4_ photosynthetic pathway is more efficient at using light, water, and nutrient resources for assimilating CO_2_ than the *C*_3_ photosynthetic pathway (Sage and Monson 1998). This is a result of biochemical and anatomical modifications that allow CO_2_ to be concentrated in bundle sheath cells of the leaf, supporting greater rates of carboxylation and lower rates of oxygenation by RuBisCO (Sage and Monson 1998; Edwards *et al.* 2010; Ermakova *et al.* 2020). Plants exhibiting *C*_4_ photosynthesis account for approximately 25% of terrestrial photosynthesis but compose only about 3% of all vascular plants (Edwards *et al.* 2010). With grasses composing approximately 60% of *C*_4_ species and capable of rapidly accumulating significant biomass, they are prime candidates for maximizing carbon acquisition and biomass allocation (Edwards *et al.* 2010; Olson *et al.* 2012).

Grass species with *C*_4_ photosynthesis such as maize (*Zea mays* L.), pearl millet [*Pennisetum glaucum* (L.) R. Br.], sorghum [*Sorghum bicolor* (L.) Moench], sugarcane (*Saccharum officinarum* L.), and switchgrass (*Panicum virgatum* L.) are among the most prominent grasses utilized in bioenergy production due to their high yields, water-use efficiency, and leaf-level nitrogen-use efficiency (Rooney *et al.* 2007; Byrt *et al.* 2011). *C*_4_ plants can achieve a higher leaf area production rate at lower leaf nitrogen levels, less fertilizer may also be used, thereby reducing nitrous oxide emissions—a major constituent of global greenhouse gases (Sage and Zhu 2011). Similarly, grasses provide the vast majority of direct and indirect calories for people worldwide (Pingali 2015), and scientists have historically targeted the mechanisms of domestication (Doebley *et al.* 2006), inflorescence improvement (Krizek and Fletcher 2005), and increased yield (Wallace *et al.* 2018), which are all entirely or partly tied to the plant’s reproductive capacity (Huang *et al.* 2016; Nadolska-Orczyk *et al.* 2017; Hunt *et al.* 2018; Juliana *et al.* 2019). However, this hyper-intense focus on plant reproductive architecture and higher harvest index in the 20th century may have precluded a more holistic understanding of sink/source dynamics and compositional components (MacNeill *et al.* 2017).

Sorghum offers excellent water-use efficiency (Enciso *et al.* 2015), nitrogen-use efficiency (Gardner *et al.* 1994), genomic simplicity (Paterson *et al.* 2009; Goodstein *et al.* 2012), phenotypic diversity (Calviño and Messing 2012), has established genomic tools and resources (Brenton *et al.* 2016; McCormick *et al.* 2018; Boyles *et al.* 2019; Mace *et al.* 2019), and sorghum can serve as a dual-purpose crop (van der Weijde *et al.* 2013). The original domestication of sorghum in the Sahel region of sub-Saharan Africa—and potentially additional domestication events (Paterson *et al.* 1997)—combined with its subsequent worldwide distribution across both latitudinal and longitudinal gradients have created immense genetic and phenotypic diversity which various cultures and communities have continued to propagate, select, and utilize for both profit and livelihood (Morris *et al.* 2013a; Lasky *et al.* 2015; Smith *et al.* 2019). This selection process has resulted in the diversification of sorghum into five botanical races (bicolor, caudatum, durra, guinea, and kafir) based on a combination of panicle architecture and seed characteristics as well as the definition of multiple types based on final process utilization (Klein *et al.* 2015). The different types may be classified based on variations in carbohydrate—*i.e.*, carbon—partitioning regimes, and these types include:

- cellulosic sorghum (carbon primarily partitioned to the stem)—originally bred in the 1920s (Vinall *et al.* 1936), again in the 1970s (Lipinsky and Kresovich 1980), and most recently in the 2010s—is mainly produced for cellulosic fibers that can be incorporated into bioenergy and bioindustrial processes (Rooney *et al.* 2007)
- forage sorghum (leaves), which is utilized in forage or silage systems for ruminant agricultural production (Bhattarai *et al.* 2019)
- grain (starch) sorghum is the most prevalent among the sorghum types and used for feed and food (Boyles *et al.* 2016; Sapkota *et al.* 2020), but may also be used in ethanol production through the conversion of starch stored in the grain (Wu *et al.* 2010)
- sweet (nonstructural sugars) sorghum is selected and bred for the extraction of simplified sugars (*i.e.*, sucrose, fructose, and glucose) in the stem (Murray *et al.* 2009; Brenton *et al.* 2020)

While differences in carbon partitioning, translocation, and storage are also common among the other grasses, the mechanisms mediating these differences on a broad scale are unclear (Vogel 2008; MacNeill *et al.* 2017; Hartmann *et al.* 2020).

In contrast to the varying ploidy levels in sugarcane (2*n* = 20 to 200), sorghum is a diploid species (2*n* = 20) with an approximately 730 Mb genome making it a simpler model for genomic research, and in contrast to maize, sweet sorghum can both yield high biomass and has been intensively bred to accumulate fermentable sugars—primarily sucrose—in their stems (Calviño and Messing 2012). Both corn and sugarcane are also very energy and water intensive, whereas sorghum’s high water-use efficiency allows it to be grown on marginal or nonarable land (Ali *et al.* 2008). The accumulation of fermentable sugars in sweet sorghum is beneficial since ethanol produced from cellulose has a higher production cost compared to ethanol produced from fermentable sugars (Calviño and Messing 2012). Sweet and cellulosic sorghums demonstrate a significant range in compositional traits and are amendable to significant modifications in carbon partitioning between structural and nonstructural carbohydrate composition (Zhao *et al.* 2009; Mullet *et al.* 2014; Brenton *et al.* 2016).

Nested association mapping (NAM) populations are a type of multiparent population generated by crossing several diverse founders with one recurrent parent (Buckler *et al.* 2009; Ladejobi *et al.* 2016). Because the chance of recombination is lower over short genetic distances and a specific number of generations, the genomes of the resulting recombinant inbred-lines (RILs) contain chromosomal segments that are a mixture of their parental genomes (Stich 2009). In contrast to diversity panels, NAM populations require a fewer number of SNPs for whole-genome scans, have higher statistical power, are less sensitive to genetic heterogeneity, and use marker information more efficiently while maintaining high-allele richness (Yu and Buckler 2006). As such, NAM populations have been used in a variety of plant systems including maize (Yu *et al.* 2008; McMullen *et al.* 2009), barley (Maurer *et al.* 2015), wheat (Bajgain *et al.* 2016), rice (Fragoso *et al.* 2017), *Brassica napus* (Hu *et al.* 2018), and sorghum (Marla *et al.* 2019).

The development of the Sorghum Carbon-Partitioning NAM (CP-NAM) population involved the collection of phenotypically and genetically diverse accessions from the Sorghum Bioenergy Association Panel (BAP) (Brenton *et al.* 2016; Flinn *et al.* 2020) such that all five of the major botanical races are represented as well as the four major types. Several of these accessions are also photoperiod sensitive. Photoperiod sensitivity is well-documented in sorghum (Quinby 1967; Major *et al.* 1990; Rooney and Aydin 1999) and is known to be regulated by at least six maturity genes, *Ma*_1_–*Ma*_6_ (Rooney and Aydin 1999). Photoperiod sensitive sorghum do not transition to reproductive growth until day lengths fall below approximately 12 h 20 min, allowing for the increased accumulation of structural and nonstructural carbohydrates (Rooney and Aydin 1999). As such, these accessions represent extremes in their ability to accumulate and partition carbon.

The recurrent parent, Grassl, was selected due to its ability to accumulate substantial biomass and fermentable carbohydrates per unit time and area (Kresovich *et al.* 1988). Grassl is also highly resistant to *Peronosclerospora sorghi* and *Puccinia purpurea*, resistant to *Sporisorium* [*Sphacelotheca*] *reiliana* and tolerant to the maize dwarf mosaic virus (Kresovich *et al.* 1988). The construction of this NAM complements the existing sorghum resources and the ongoing reference genome assemblies, pan-genomics projects, and database development which should increase the utility and accessibility for researchers worldwide (Boyles *et al.* 2019). The incorporation of photoperiod sensitive, nontemperately adapted material provides germplasm that is not confounded by the prevalence of dwarfing and photoperiod insensitive alleles (Wang *et al.* 2020). Here, we perform a quality assessment of the CP-NAM as a genomic resource and validate the population for use in genomic studies using pericarp and testa phenotypes as positive controls to map known genes affecting these phenotypes.

## Materials and methods

### Plant materials and phenotyping

The CP-NAM parents were grown in Florence, South Carolina, at the Clemson University Pee Dee Research and Education Center in 2013 and 2014 with two complete randomized blocks planted each year as a part of work done by Brenton *et al.* (2016). As mature plant height exceeded irrigation pivot height in many accessions, irrigation was halted approximately 90 days after planting. Seed treatment was performed as described in Brenton *et al.* (2016). The selected traits included anthesis date, stalk weight, leaf weight, panicle weight, juice weight, brix, wet weight, dry weight, total weight, plant height, acid detergent fiber, neutral detergent fiber, nonfibrous carbohydrates, lignin, and water-soluble carbohydrates (Table 2 and Supplementary Table S1) (Brenton *et al.* 2016). Plant height was measured at physiological maturity or harvest from the stalk base to either the panicle apex or the shoot apical meristem apex in the event a panicle did not develop. Samples were dried at 40 °C to a constant weight before measuring dry weight. Compositional data were generated from dried samples using a Perten DA7250 near-infrared spectroscopy (NIR) instrument (https://www.perten.com) as described in Brenton *et al.* (2016). The CP-NAM parent PI329311 is not represented in the agronomic data due to an inability to acquire sufficient germplasm.

**Table 2:** CP-NAM parent agronomic and physiological traits

Accession*^a^*	Days to Harvest	Stalk weight (kg)	Leaf weight (kg)	Panicle weight (kg)*^b^*	Brix	WSC (%DM)
PI154844	163.25 ± 5.19	2.57 ± 1.01	0.39 ± 0.15	0.14 ± 0.07	12.95 ± 1.67	−
PI22913	122.67 ± 2.31	1.31 ± 0.4	0.17 ± 0.04	0.14 ± 0.03	13.9 ± 0.95	27.4
PI229841	136 ± 18.97	1.05 ± 0.26	0.19 ± 0.07	0.24 ± 0.06	10.35 ± 3.41	16.48 ± 3.33
PI297130	158.5 ± 4.95	2.59	0.43 ± 0.1	−	11.8 ± 0.99	24.92 ± 1.8
PI297155	114 ± 7.66	0.56 ± 0.08	0.16 ± 0.08	0.14 ± 0.06	6.33 ± 1.55	−
PI506069	159 ± 7.07	2.13	0.44 ± 0.11	−	7.9 ± 0.28	10.03 ± 5.3
PI508366	156	2.38	0.4 ± 0.1	−	6.35 ± 0.07	12.1 ± 3.61
PI510757	157 ± 2.83	2.56	0.55 ± 0.01	−	7.15 ± 2.9	25.91 ± 1.55
PI563295	138.25 ± 14.34	1.87 ± 0.6	0.24 ± 0.07	0.16 ± 0.08	14.38 ± 3.55	31.84 ± 9.51
PI586454	114.25 ± 8.42	1.18 ± 0.31	0.2 ± 0.05	0.12 ± 0.04	11.73 ± 1.86	−
PI655972	110.5 ± 3.7	0.49 ± 0.03	0.14 ± 0.04	0.13 ± 0.07	8.78 ± 2.35	9.5 ± 2.32

Cells contain the mean ± the standard deviation for each trait where replicate data were available. Single-replicate data do not contain standard deviations, and missing data are represent by “−.”

a PI329311 did not have adequate germplasm for inclusion.

b Photoperiod sensitive lines did not produce panicles.

A total of 11 RIL families were generated using diverse sorghum lines and female Grassl (Table 1), which resulted in approximately 200–274 individuals for each RIL family between diverse sorghum lines including bicolor, caudatum, durra, guinea, kafir, kafir-bicolor, and female Grassl (Table 3). Plant accessions were obtained through the Agricultural Research Service-Germplasm Resources Information Network (ARS-GRIN) (http://www.ars-grin.gov). Families derived from PI329311 and PI510757 were crossed in Puerto Vallarta, Mexico in the winter of 2012. All other crosses were made in the winter of 2013. The *F*_1_s were grown out in Florence, South Carolina the following summers—2012 for the two families above and 2013 for the rest. Subsequent generations were grown in Puerto Vallarta each year. The panicles of each generation were bagged to prevent outcrossing and ensure selfing. The *F*_6_ RILs were phenotyped for testa pigmentation and pericarp color where three seeds were selected to represent each line. Pericarp color was visually assessed and categorized as red, brown, yellow, or white (Supplementary Figure S1). Subsequently, the grain was split with a razor blade, and the testa was visually inspected for the presence or absence of pigmentation (Choi *et al.* 2019).

**Table 1:** NAM parent characteristics

Common name	Accession	Race	Origin	Sorghum type	Pericarp*^a^*	Testa*^b^*
Grassl	PI154844	Caudatum	Uganda	Cellulosic	r	y
Chinese Amber	PI22913	Bicolor	China	Sweet	b	y
IS 2382	PI229841	Kafir	South Africa	Grain	r	y
IS 13613	PI297130	Caudatum	Uganda	Cellulosic	w	y
IS 13633	PI297155	Kafir	Uganda	Grain	r	y
IS 11069	PI329311	Durra	Ethiopia	Cellulosic	y	n
Mbonou	PI506069	Guinea-bicolor	Togo	Cellulosic	y	n
MA 38	PI508366	Guinea	Mali	Cellulosic	w	n
AP79-714	PI510757	Durra	Cameroon	Cellulosic	w	n
Rio	PI563295	Durra-caudatum	Maryland, USA	Sweet	w	y
Leoti	PI586454	Kafir-bicolor	Hungary	Sweet	r	y
Pink Kafir	PI655972	Kafir	Kansas, USA	Forage	w	n

The characteristics of each NAM parent include the common name, USDA plant introduction numbers, botanical race, original source of germplasm, type as defined in the introduction, pericarp color, and presence or absence of pigmentation within the testa layer.

a b, brown; r, red; y, yellow, and w, white.

b y, pigmented and n, not pigmented.

**Table 3:** RIL family statistics

Male parent	No. indiv.	No. markers	Avg. inbreeding coefficient
PI22913	203	9,258	0.62
PI229841	209	7,422	0.60
PI297130	243	8,770	0.60
PI297155	216	7,470	0.61
PI329311	240	9,460	0.58
PI506069	204	7,756	0.59
PI508366	232	7,097	0.63
PI510757	274	7,193	0.58
PI563295	245	6,656	0.56
PI586454	200	8,038	0.57
PI655972	223	7,430	0.63

The columns from left to right are the male parent plant introduction number (column 1), the total number of individuals (column 2), markers in each family (column 3), and the average inbreeding coefficient (column 4).

### Genotype-by-sequencing data production and processing

Genotyping by sequencing (GBS) data were generated at the University of Wisconsin using leaf tissue collected from 2-week-old seedlings for each individual at the *F*_6_ generation. DNA was extracted using a modified CTAB protocol and double-digested using the enzymes *Pst*I and *Msp*I, which improve the fidelity of SNP markers, are better at reducing genomic complexity and generate a more uniform library than *Ape*KI (Poland *et al.* 2012; Thurber *et al.* 2013). GBS libraries were single-end sequenced using an Illumina HiSeq2500 sequencer except for one plate which was sequenced on a NovaSeq6000 resulting in 100-bp reads. GBS were processed using Tassel version 5.2.52 (Bradbury *et al.* 2007) following the GBS version 2 pipeline procedures (Glaubitz *et al.* 2014). Tags were aligned to the BTx623 version 3.1 annotated reference genome (McCormick *et al.* 2018), obtained from Phytozome (Goodstein *et al.* 2012), using BWA version 0.7.17 (Li and Durbin 2010). Beagle version 5.1 was used to impute missing genotype data in the variant call format (VCF) file resulting from the Tassel pipeline (Browning *et al.* 2018). Prior to mapping, SNPs were pruned using Plink (–indep 50 5 2) to reduce the number of associations derived from SNPs within LD blocks.

SNP density plots were generated using R-CMplot version 3.6.0 (https://github.com/YinLiLin/R-CMplot). The inbreeding coefficient, nucleotide diversity and Tajima’s D were calculated on family-specific VCFs using VCFtools version 0.1.16 (Danecek *et al.* 2011). Nucleotide diversity and Tajima’s D (–window 100000) were plotted in R (R Core Team 2019) by chromosome for each family (Supplementary Figures S6 and S7). The effects of SNPs were predicted using snpEff (Cingolani *et al.* 2012) and plotted using MultiQC (Ewels *et al.* 2016), and linkage disequilibrium (LD) statistics were calculated using Plink v1.90b6.10 (Purcell *et al.* 2007). The LD decay plot was generated using PopLDdecay (Zhang *et al.* 2019) with a 300 kb window and custom R scripts were LD decay was estimated for individual chromosomes as well as genome-wide (Hu *et al.* 2019). The sorghum reference genome was also *in silico* digested using restriction sites for the enzymes *Pst*I (CTGCA—G) and *Msp*I (C—CGG) using a custom R script adapted from Hu *et al.* (2019), and the segment lengths from the digestion were obtained using the R package SimRAD (Lepais and Weir 2014) and plotted using a custom CPython script (Van Rossum and Drake 2009) and the package seaborn (Waskom *et al.* 2017).

### NAM population contrast and structure

Data for the Tx430 grain NAM was accessed from Dryad (Hu *et al.* 2019) and filtered using the individuals unique to the Tx430 grain NAM (Bouchet *et al.* 2017). The variants for both the Tx430 grain and CP-NAM had the reference alleles corrected using a custom script (https://github.com/jlboat/CP-NAM) before merging the populations using VCFtools (Danecek *et al.* 2011). The merged variants were filtered using VCFtools for <20% missing data, and the common SNPs were used to assess genotypic diversity between the two populations. Principal component analysis (PCA) was performed on the individual and merged populations using SNPRelate (Zheng *et al.* 2012). In the CP-NAM PCA plot, Grassl is represented by “x” for clarity.

Population structure was estimated from the pruned SNPs using ADMIXTURE (Alexander and Lange 2011). Fivefold cross-validation was used to determine the optimal number of ancestral populations, K, by selecting the model that had the lowest cross-validation error (*K* = 15; Supplementary Figure S11). The Q matrix of the selected model—representing the ancestry fractions of individuals—was then sorted by ancestry coefficient for each subpopulation such that individuals with coefficients >50% were assigned to the corresponding subpopulation. Subpopulations were classified as Q1–Q15 as determined by the column containing the sorted ancestry coefficient. This classification was used to represent ancestral admixture of individuals in the CP-NAM PCA.

### Quantitative trait loci mapping

The imputed genotype matrix was filtered to create a separate VCF file for each RIL family using VCFtools (Danecek *et al.* 2011). Variants with minor allele frequency (MAF) <0.05 and missing data (>0.3) were removed from each family using VCFtools before converting RIL genotypes to ABH format using Genotype-Corrector (Miao *et al.* 2018), where A and B alleles were derived from parents A and B, respectively, and H represents a heterozygous marker call. Pseudomarkers were inserted into the genetic map at 1 cM intervals prior to calculating conditional genotype probabilities. The conditional probabilities of the true genotypes were estimated using a hidden Markov model for each RIL family with a genotyping error rate of 0.0001 and Haldane’s mapping function (Kosambi 2016). Quantitative trait loci (QTL) were mapped for each RIL family using both Haley-Knott regression (Haley and Knott 1992) and a linear mixed model accounting for relationships among individuals using a random polygenic effect using R qtl2 version 0.22 (Broman *et al.* 2019). Kinship matrices were calculated using the allele probabilities and incorporated into a linear mixed model genome scan performed using along with pericarp phenotypic data (Broman *et al.* 2019). Due to reduced noise in the linear mixed model results, Haley-Knott regression results are not discussed.

### Genome-wide association studies

The software GEMMA version 0.98.1 (Zhou and Stephens 2014) was used for genome-wide association studies (GWAS). The imputed VCF file containing the entire NAM was converted to Plink format using VCFtools (Danecek *et al.* 2011) before using Plink (Purcell *et al.* 2007) to generate the accompanying phenotype files. GEMMA was then used to calculate a standardized relatedness matrix—where the genotype data are standardized before estimating a relatedness matrix (Astle and Balding 2009)—for linear mixed modeling on the filtered data (–miss = 0.3 –maf = 0.05) (Zhou and Stephens 2014). Models were initially run with principal components (PCs) as covariates. However, the inclusion of PCs did not alter associated variants. As a result, all models described did not contain PCs. Univariate and multivariate models were run to determine the effects of testa pigmentation on the mapping of pericarp color. Where univariate linear mixed models were fit using the following form:

$$\begin{array}{c} y=W\alpha +x\beta +u+\epsilon ;\\ u∼MVN_{n}(0,\lambda \tau^{-1}K), \end{array}$$

where **y** is a vector of pericarp colors for *n* individuals; **W** is a matrix of covariates including a column of 1 s for estimating the intercept; α is a vector of the corresponding coefficients; *x* is an *n*-vector of genotypes; *β* is the effect size of the marker; **u** is a vector of random effects; ϵ is a vector of errors; $\tau^{-1}$ is the variance of the residual errors; *λ* is the ratio between the two variance components; **K** is a known *n *×* n* standardized relatedness matrix (Zhou and Stephens 2014).

Models included a univariate model using pericarp color with all phenotypes, a univariate model with binary encodings for the yellow phenotype—where yellow is 1 and all other phenotypes are 0, a univariate pericarp model with testa pigmentation as a covariate, a univariate model with pericarp color and testa pigmentation covariate based on three pericarp colors (red, yellow, and white), and a multivariate model using both pericarp color and testa pigmentation. Where the multivariate linear mixed model was fit using the following form:

$$Y=WA+x\beta^{T}+U+E;$$

where **Y** is a *n *×* d* vector of *n* individuals and *d* phenotypes—namely pericarp color and testa pigmentation; **W** is a vector of 1 s to estimate the intercept; **A** is a matrix of the corresponding coefficients; **x** is an *n*-vector of genotypes; *β* is a *d*-vector of marker effect sizes for the *d* phenotypes; **U** is an *n *×* d* matrix of random effects; and **E** is an *n *×* d* matrix of errors (Zhou and Stephens 2014). Manhattan and Q-Q plots were generated using R-CMplot version 3.6.0 (https://github.com/YinLiLin/R-CMplot).

### Data availability

Raw GBS data are available at the European Nucleotide Archive under the project accession PRJEB40592. The Tx430 NAM data were accessed from dryad at doi: 10.5061/dryad.63h8fd4 (Hu *et al.* 2019). Scripts are available on GitHub (https://github.com/jlboat/CP-NAM) under MIT License. Code freezes are available for BWA and PopLDdecay via Singularity containers (Kurtzer *et al.* 2017) and were executed using Singularity version 3.5.3. Containers may be directly pulled from SingularityHub: https://singularity-hub.org/collections/2877. Supplemental materials available at figshare: https://figshare.com/s/0ba752156d0cb7fb6404. CP-NAM seeds are available upon request.

## Results

### CP-NAM parent carbon-partitioning diversity

The CP-NAM parents were selected due to the broad phenotypic variance of their carbon-partitioning traits (Table 2 and Supplementary Table S1). The selected traits capture the primary above-ground carbon-partitioning regimes represented by the major sorghum types. The overall distribution of each phenotype was largely consistent across years and replicates (Supplementary Figure S2), and the phenotypic distributions across all accessions was quite broad (Supplementary Figure S3). Due to the photoperiod sensitivity of many of the parental accessions, significant quantities of structural and carbohydrates are accumulated across both stems and leaves, and appreciable nonstructural carbohydrates are stored within the stems (Table 2 and Supplementary Table S1). Accessions that did not flower (*i.e.*, photoperiod sensitive accessions) were not included in the panicle weight data since they never produced panicles.

### *In silico* digestion and single-nucleotide polymorphisms

**Figure 1:**
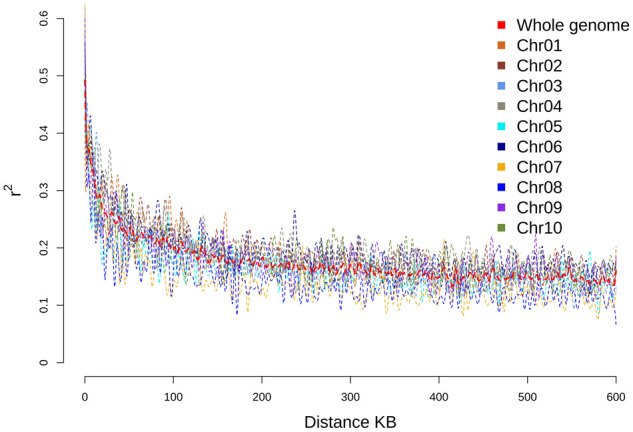
Linkage decay (Pearson’s correlation coefficient squared) plotted against the distance in kilobases across the genome.

The complete NAM population genotypic data contained 144,087 SNPs after imputation of the original SNP calls, which corresponds to an average density of one SNP per 5 kb. The subtelomeric SNP density was higher than the pericentromeric regions (Supplementary Figures S4 and S5). To assess the putative restriction sites for *Pst*I and *Msp*I restriction enzymes, the BTx623 reference genome was *in silico* digested (Supplementary Figures S6 and S7), and as seen with SNP density, restriction sites were primarily concentrated in subtelomeric regions with gaps surrounding centromeric regions. The patterns of nucleotide diversity were largely consistent across families with greater diversity seen around centromeres (Supplementary Figure S8). Similarly, patterns of Tajima’s D statistics were more variable around centromeres and typically positive across all families and chromosomes except for some regions showing strong directional selection (Supplementary Figure S9). The effects of all SNPs were analyzed using snpEff (Cingolani *et al.* 2012) and plotted using MultiQC (Ewels *et al.* 2016) (Supplementary Data 1). The overall transition-transversion ratio was 1.649 with about half of the variant effects occurring upstream or downstream of known loci, approximately 20% of effects falling into intergenic regions, and the remaining 30% occurring within genic regions. The impacts of most effects were predicted to be modifier effects (85%) with the remaining effects distributed approximately equally among low, moderate, and high impact (5% each). The LD decay was estimated for individual chromosomes as well as genome-wide (Figure 1). The genome-wide LD decays to *r*^2^ < 0.2 around 100 kb, and Chr 6 exhibits consistently higher LD compared to the other chromosomes.

### Validating RIL families

Pruning reduced the number of SNPs from 144,087 to 57,603, and after filtering SNPs with a MAF < 0.05, that number was further reduced to 38,682. Marker counts for individual families ranged from about 7000 to over 9000, and the average inbreeding coefficients were approximately 0.6 for all families (Table 3). Among the RILs, all families had at least one QTL for pericarp color except for the PI229841, PI297155, and PI586454 RILs (Supplementary Tables S2 and S3; Supplementary Figures S29–S39). Most families had a QTL located on Chr 4 (62.4 Mb), but there were also hits on Chr 1 (PI508366 and PI563295 RILs), two (PI508366 RILs), seven (PI297130 and PI506069 RILs), and nine (PI655972 RILs). Notably, the PI563295 RILs had a significant QTL spanning 23 Mb on Chr 1 for pericarp color. Because QTL mapping results closely overlapped GWAS results, all genetic mapping results discussed henceforth with be based on GWAS results.

### NAM population contrast and structure

**Figure 2:**
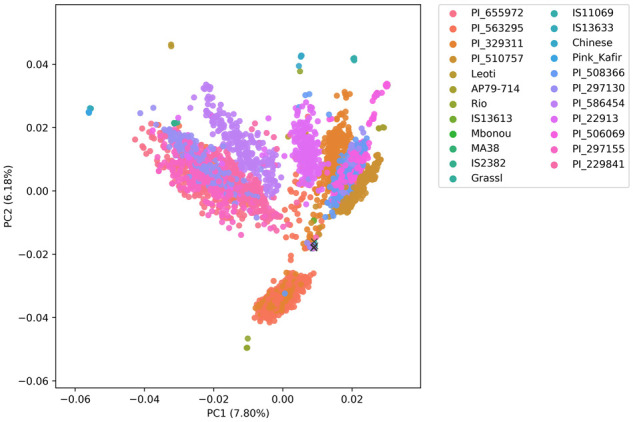
Principal component analysis plot using the whole CP-NAM population. Parents are labeled with the common name. The individual samples of the recurrent parent, Grassl, are additionally labeled with “x.” Each RIL family is represented by the male parent PI.

For further quality control, a PCA was performed for the CP-NAM. As expected, RILs were oriented toward their corresponding parents, which is indicative of the genetic mosaicism within these lines (Figure 2). The first 10 PCs account for 34.7% of the genomic variation with the first two PCs explaining 7.80 and 6.18% of the variation, respectively. Similarly, a PCA was conducted using the set of common SNPs (8289) between the Tx430 grain NAM and the CP-NAM (Supplementary Figure S10) to compare the genetic differences between the two populations. Substantial variation was observed both within and between the NAM populations (Supplementary Figures S11 and S12). The first PC clearly separated the two NAM populations and accounted for 19% of the variance—substantially higher than the 7.5% for the first PC of individual NAM populations—while the second PC accounted for 5% of the variance (Supplementary Figure S11). The distribution of common SNPs between the two NAM populations was similar across the genome (Supplementary Figure S10) as compared to the distribution within the CP-NAM alone (Supplementary Figures S4 and S5).

**Figure 3:**
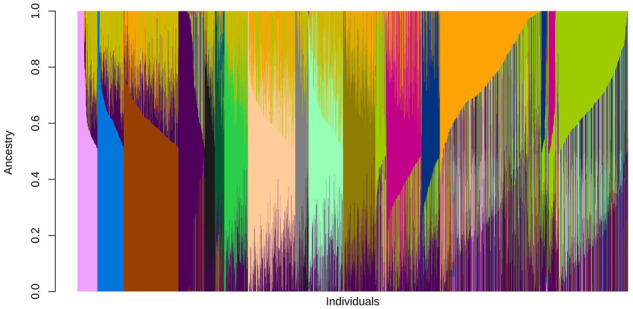
Genome-wide, population admixture of the CP-NAM. Individuals (*x*-axis) are shown as vertical bars colored in proportion to their estimated ancestry within each cluster (*y*-axis) based upon 15 ancestral populations (*K* = 15) where each genetically distinct ancestral population is given a unique color.

**Figure 4:**
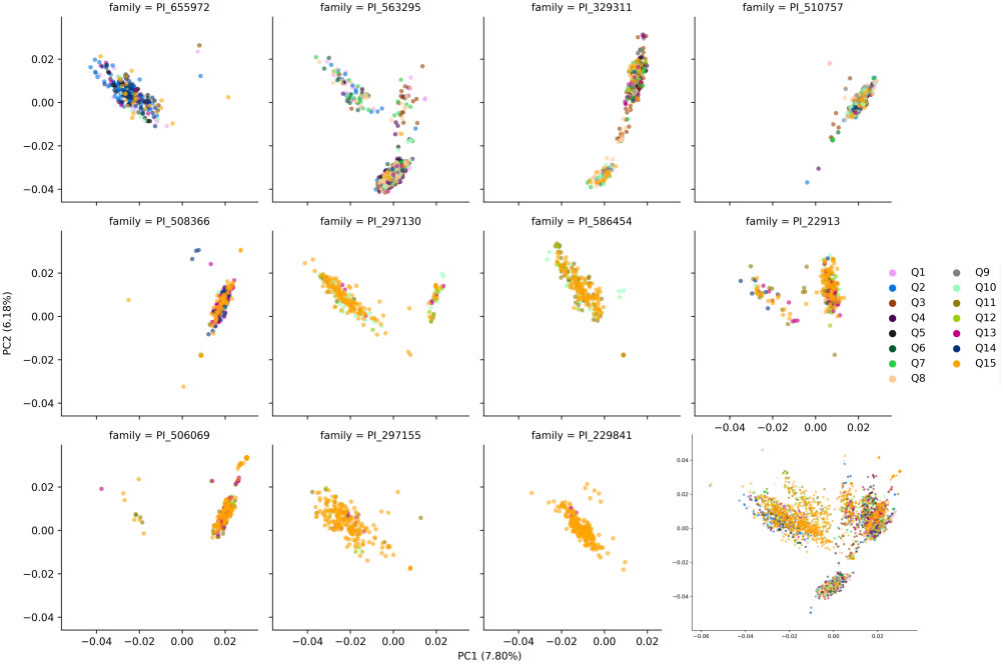
CP-NAM PCA with admixture coloration. Individuals were classified as Q1–Q15 as determined by the proportion of ancestral admixture. Cells 1–11 represent individual RIL families represented by the paternal identifier while the 12th cell contains the entire CP-NAM population.

Analysis of population admixture resulted in the identification of 15 ancestral populations within the CP-NAM (Figure 3). Multiple subgroups were identified within the major sorghum botanical races. When the ancestral population classification was superimposed over the CP-NAM PCA, admixture among RIL families is clearly identifiable (Figure 4).

### Genome-wide association studies

#### Univariate GWAS for pericarp color

**Figure 5:**
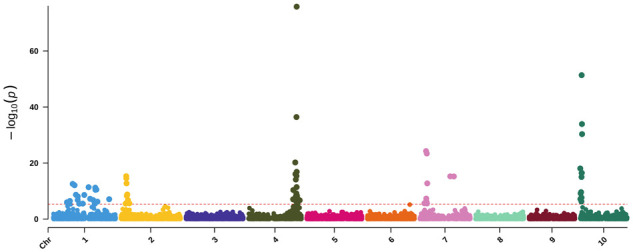
Univariate GWAS for pericarp pigmentation. The −log10 *P*-values (*y*-axis) are plotted against the position on each chromosome (*x*-axis). Each circle represents a SNP, and the red dashed line represents the Bonferroni-corrected threshold.

The GWAS for the entire NAM population identified six significant peaks across four chromosomes. Chromosome one contained a single broad peak—also identified by the PI563295 RILs QTL mapping—that was somewhat resolved by binary encoding for yellow pericarp (as described below). Two hits were identified on Chr 2 (at 6,940,113 and 57,797,411), and the most significantly associated SNP was near 62 Mb on Chr 4 (62,215,490 bp; 3.76E-31). There were also two hits on Chr 7 (9,097,206 and 44,198,228 bp) (Figure 5 and Supplementary Figure S14). We also mapped all pericarp colors with testa as a covariate to account for the spread of tannin from the testa layer and the traditional (red, yellow, and white) colors, but the differences were minimal compared to three GWAS discussed here (Supplementary Figures S15 and S16).

#### Univariate GWAS for yellow pericarp

**Figure 6:**
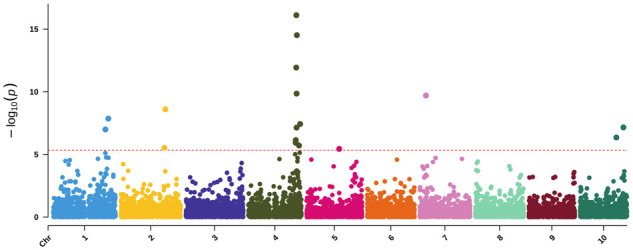
Univariate GWAS for binary encoding of yellow pericarp pigmentation. The −log10 *P*-values (*y*-axis) are plotted against the position on each chromosome (*x*-axis). Each circle represents a SNP, and the red dashed line represents the Bonferroni-corrected threshold.

Since the GWAS containing all phenotypes failed to identify the *yellow seed1* (*y1*) locus (*Sobic.001G397900*), the phenotypes were then given binary encodings for yellow pericarp color. With binary phenotypes for yellow pericarp color, we were able to identify a peak near *y1* (Figure 6, Supplementary Figure S18; Table 4) and increase the significance for the second peak on Chr 2 (57,797,411 bp) identified in the standard encoding GWAS. The hit on Chr 4 is the same SNP as that for the univariate pericarp mapping. The SNP on Chr 7 (9,097,206 bp) mapped in the univariate GWAS for all pericarp colors is within a 1 Mb proximity of the SNP on chromosome seven (8,111,484 bp) mapped here and highly correlated (*r*^2^ = 0.83). The additional SNP on Chr 10 (56,346,032 bp) falls within a QTL (56,223,543–56,564,728 bp) previously identified when mapping endosperm carotenoid content—though the exact gene regulating the trait is unclear (Fernandez *et al.* 2008; Mace *et al.* 2019).

**Table 4:** Top associations from the NAM GWAS

Model	Chromosome	Position	Wald *P*-value	Genes
Univariate Pericarp	1	−	−	<block too large>
Univariate Pericarp	2	6,940,113	1.23E-10	tan2
Univariate Pericarp	2	57,797,411	2.26E-06	Classical Z locus; Sobic.002G190000
Univariate Pericarp	4	62,215,490	3.76E-31	tan1
Univariate Pericarp	7	9,097,206	4.86E-15	Unknown
Univariate Pericarp	7	44,198,228	2.66E-08	Unknown
Univariate Yellow	1	71,320,809	1.39E-08	y1
Univariate Yellow	2	57,797,411	2.50E-09	Classical Z locus; Sobic.002G190000
Univariate Yellow	4	62,215,490	7.70E-17	tan1
Univariate Yellow	7	8,111,484	2.00E-10	Unknown
Univariate Yellow	10	56,346,032	6.96E-08	Putative carotenoid regulator
Multivariate Pericarp	1	−	−	<block too large>
Multivariate Pericarp	2	6,940,113	5.00E-16	tan2
Multivariate Pericarp	4	62,215,490	3.82E-37	tan1
Multivariate Pericarp	4	62,463,940	1.55E-76	tan1
Multivariate Pericarp	6	55,070,387	6.89E-06	Putatively *tt16* ortholog
Multivariate Pericarp	7	8,111,484	5.54E-25	Unknown
Multivariate Pericarp	7	39,531,969	5.51E-16	Unknown
Multivariate Pericarp	7	44,198,228	6.02E-16	Unknown
Multivariate Pericarp	10	1,948,816	4.30E-52	*waxy*

For each significant association, the model, chromosome containing the SNP, SNP position, wald *t*-test *P*-value, and putative gene in LD with the significant SNP are identified.

#### Multivariate GWAS for pericarp color and testa pigmentation

**Figure 7:**
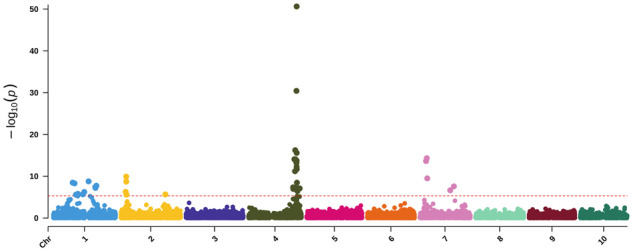
Multivariate GWAS for pericarp color and testa pigmentation. The −log10 *P*-values (*y*-axis) are plotted against the position on each chromosome (*x*-axis). Each circle represents a SNP, and the red dashed line represents a Bonferroni-corrected threshold.

Multivariate GWAS for the entire NAM population had peaks similar to those identified in the univariate analyses (Figure 7; Table 4) with the exception of novel peaks on chromosomes six and 10. The peak on Chr 6 (55,070,387 bp) is close to a known QTL (55,653,174–55,805,785 bp) mapped using brown grain pigmentation (Rhodes *et al.* 2014). The multivariate GWAS identified two SNPs around 42 Mb on Chr 7, one of which is the same SNP as the univariate GWAS (Chr7:44,198,228 bp). These two SNPs were highly correlated with each other (*r*^2^ = 0.97) even though there is approximately 5 Mb between them. Last, a peak at Chr10 (1,948,816 bp) was identified, which was unique to the multivariate analysis.

## Discussion

Sorghum was domesticated around 5,000 years ago and has since become a major cereal crop and the primary crop of arid zones (De Wet and Harlan 1971; Smith *et al.* 2019). Sorghum has historically been valued as a crop for its stem sugars and grain (Wall and Blessin 1970; Subramanian *et al.* 1987)—with improvement focusing on a single nonstructural carbohydrate either sucrose or starch, respectively (Murray *et al.* 2008; Saballos 2008) and more recently as a source of biomass (Murray *et al.* 2008). In contrast to some other *C*_4_ grains and grasses, sorghum production can be successful in low- and high-input agriculture due to its ability to grow in marginal soils, and it serves as a dual-purpose crop due to the production of both grain and stem sugars (Calviño and Messing 2012). The diverse carbon-partitioning regimes of sorghum provide a unique opportunity to study the processes underlying source-sink relationships in the *C*_4_ grasses. In addition, the genetic properties of sorghum such as its compact, diploid genome (730 Mb) (Paterson *et al.* 2009), the availability of genetic and genomic resources (Brenton *et al.* 2016; McCormick *et al.* 2018; Boyles *et al.* 2019; Mace *et al.* 2019) and broad phenotypic variability (Morris *et al.* 2013a) further establish sorghum as a vital crop for not only bioenergy (Calviño and Messing 2012) but also carbon partitioning more generally (Slewinski 2012).

As the cost of developing genomic resources has continued to decline, sorghum has seen an increase in publicly available genomic resources (Boyles *et al.* 2019). Genetic mapping populations are designed to effectively dissect the genomic architecture underlying specific traits, and sorghum mapping population resources include diversity panel (Morris *et al.* 2013a; Brenton *et al.* 2016), grain NAM (Bouchet *et al.* 2017; Marla *et al.* 2019), multiparent advanced generation intercross (Ongom and Ejeta 2018) and mutagenized populations (Xin *et al.* 2008; Addo-Quaye *et al.* 2018). As the number of available populations increases, newly established populations should demonstrate unique utility, whether that be easier management, improved throughput, a specific phenotypic focus, or better statistical design. For instance, all four, alternative sorghum NAMs focus on grain sorghum (Boyles *et al.* 2019), and the CP-NAM uniquely allows for the genetic dissection of pathways facilitating carbon-partitioning regimes that may be exploited for bioenergy traits as well. The CP-NAM parents were selected from the BAP to maximize the phenotypic and genetic diversity of their carbon-partitioning traits. Preliminary phenotypic characterization of the parents was done as a part of Brenton *et al.* (2016). These phenotypes covered a variety of agronomic and physiological traits (Supplementary Figures S2 and S3; Table 2 and Supplementary Table S1) and demonstrate substantial diversity among the CP-NAM parents. In particular, the CP-NAM parents are able to accumulate significantly more structural and nonstructural carbohydrates than grain sorghum due to the inclusion of photoperiod sensitive lines (Rooney and Aydin 1999; Brenton *et al.* 2016). The accumulation of carbon is also variable across the parts of the plants with parental accessions differentially allocating carbon to the various sinks (Table 2).

To further distinguish this population, we provide a comparison of the CP-NAM to an existing, publicly available sorghum grain NAM population developed with RTx430 as the recurrent parent (Bouchet *et al.* 2017). We selected Tx430 NAM because it was not a backcross NAM as two of the existing NAM populations are, and of the two remaining sorghum NAMs, Tx430 was the only population with publicly available variants at the time of writing. These NAM populations were both sequenced using GBS and share 8289 SNPs in common with <20% missing data (Supplementary Figures S10 and S11). While the Tx430 grain NAM used *Ape*KI restriction digest, we utilized a double digest using the enzymes *Pst*I and *Msp*I, which improve the fidelity of SNP markers, are better at reducing genomic complexity and generate a more uniform library than *Ape*KI (Supplementary Figures S6 and S7) (Poland *et al.* 2012; Thurber *et al.* 2013). The two populations are both genotypically diverse (Supplementary Figure S11) and yet distinct as the first principal component (PC1), which explains over 19% of the variation, effectively separates the two populations. The CP-NAM also exhibits significant diversity across families as demonstrated by nucleotide diversity (Supplementary Figure S8), Tajima’s D (Supplementary Figure S9), and PCA (Figure 2).

Population structure was also assessed to determine the degree of ancestral genetic admixture captured by the CP-NAM (Figure 3). Cross-validation identified 15 major ancestral populations represented in the CP-NAM with notable admixture occurring even within RIL families (Figure 4). This admixture is evident across different sorghum races and the major sorghum types—revealing that the CP-NAM captures a notable portion of sorghum diversity. The RIL families from PI329311, PI510757, PI563295, and PI655972, exhibit the strongest degree of population subdivision relative to the other RILs and represent three of the four major sorghum types. RILs derived from paternal bicolor races (*i.e.*, PI22913 and PI506069) also demonstrate higher admixture than RILs derived from other races such as kafir (*i.e.*, PI229841 and PI229155), which is known to be geographically limited and exhibits stronger genetic bottleneck (Deu *et al.* 2006; Klein *et al.* 2008; Sapkota *et al.* 2020). The representation of historical admixture within the CP-NAM permits the elucidation of the mechanisms regulating carbon partitioning among the various sorghum types since their initial divergence. As LD influences the resolution at which we can identify trait mapping and informs breeding decisions, the LD decay was estimated for individual chromosomes as well as genome-wide (Figure 1). The genome-wide LD decays to *r*^2^ < 0.2 around 100 kb, and Chr 6 exhibits consistently higher LD compared to the other chromosomes, which is consistent with previous findings concerning limited recombination on Chr 6 (Hu *et al.* 2019) and a high degree of synteny between sorghum and *Oryza sativa* L. (Kim *et al.* 2005).

Sorghum pericarp and testa pigmentation are well-characterized domestication traits, which are regulated by a few loci (Zhang *et al.* 2015) and therefore serve as good quality-control targets for genetic validation of new genomic resources (Morris *et al.* 2013b). Sorghum seed color phenotypes vary based upon carotenoid and polyphenol compounds present within the corresponding kernel layers (Rhodes *et al.* 2014). The primary pericarp colors—red, yellow, and white—are regulated by the *R* and *y1* loci, but due to additional loci that further modulate pericarp color such as I (intensifier), S (spreader), and Z (mesocarp thickness), pericarp color also comes in black, brown, orange, and pink as well as ranges of those colors varying in tint, shade or even spotted (Dykes *et al.* 2013; Rhodes *et al.* 2014).

Condensed tannins—a subtype of polyphenol—strongly contribute to kernel pigments in sorghum grain and are regulated by two loci—traditionally known as B1 and B2 but recently identified as, which corresponds to *tannin 1* (*tan1*) [*Sobic.004G280800*; (Wu *et al.* 2012)] and *tannin 2* (*tan2*) [*Sobic.002G076600*; (Wu *et al.* 2019)] – with duplicate recessive interaction. When either locus contains homozygous recessive alleles, condensed tannins fail to accumulate within the sorghum grain (Wu *et al.* 2019) which otherwise confer a brown pigmentation to grains. Brown pericarp, in particular, is associated with significantly higher proanthocyanidin concentrations (Rhodes *et al.* 2014) and may be used to predict the nutritional value of sorghum grains since brown seed color is associated with anti-nutritive compounds such as tannins which also confer a bitter taste (Sedghi *et al.* 2012; Ebadi *et al.* 2019). Similarly, the *y1* locus encodes a MYB family transcription factor that regulates phlobaphene—another phenolic compound—biosynthesis (orthologous to Arabidopsis *tt2*) yielding a yellow pericarp while loss of function confers a white color (Ibraheem *et al.* 2010; Rhodes *et al.* 2014), and the *R* locus confers a red tint to the pericarp but only with dominant *y1* (Doggett 1987).

By employing a variety of phenotypic encodings as well as univariate and multivariate GWAS, we were able to identify a number of these well-established loci as well as three additional loci. The univariate pericarp color GWAS (Figure 5) resulted in six peaks across four chromosomes. The lack of distinct peaks on Chr 1 has been previously observed when mapping 3-deoxyanthocyanid concentrations—associated with grain pigmentation (Rhodes *et al.* 2014)—and pericarp color where precise mapping of the *y1* gene, in particular, can prove difficult (Morris *et al.* 2013b). The two hits for pericarp color on Chr 2 likely correspond to (*tan2*) (*Sobic.002G076600*) for the first SNP (Mace and Jordan 2010; Wu *et al.* 2019) while the hit around 58 Mb—previously identified by Rhodes *et al.* (2014) when mapping grain color and Hu *et al.* (2019) using mesocarp thickness—corresponds to *Sobic.002G190000*. Here, mapping of binary phenotypes based on yellow pericarp color circumvents issues associated with brown phenotypes that simply removing brown failed to resolve. Brown pericarp is known to mask the expression of *R* and *y1* genes—located on chromosomes three and one, respectively—because the phenotype is generated by the spread of tannin from the testa layer (Morris *et al.* 2013b). As such, we additionally performed a multivariate GWAS with both pericarp and testa pigmentation (Figure 7). However, we were unable to map the *R* locus—possibly due to the potentially complicating pericarp phenotypes present in this population.

Using a multivariate GWAS for both pericarp and testa color, we were able to identify *tan1* and *tan2* loci (Figure 7). Both loci along with the region near Chr10:56,346,032 previously associated with carotenoid content, *Sobic.006G213900* on Chr 6 and *y1* regulate polyphenolic compounds, and most of these genes are known components of the flavonoid pathway (Rhodes *et al.* 2014). The presence of these compounds directly affects kernel color. *Sobic.006G213900* encodes a MADS-box transcription factor orthologous to Arabidopsis *transparent testa 16* (*tt16*), while the peak at Chr10 (1,948,816 bp) may correspond to *waxy* (*Sobic.010G022600*), which encodes a glycosyl-transferase orthologous to Arabidopsis *granule-bound starch synthase 1* (Figure 7). All GWAS had a peak for *tan1*, which commonly occurs due to the strong effect of tannin content on pericarp color (Wu *et al.* 2012). Similarly, identification of *Sobic.002G190000—*a gene encoding a zinc-finger protein that colocalizes with the classical Z locus, which is known to regulate mesocarp thickness (Wu *et al.* 2019)—is consistent with the impact of mesocarp thickness on perceived kernel color (Mace and Jordan 2010; Hu *et al.* 2019). Both locations on Chr 7 were previously associated with inflorescence traits with the earlier peak (Chr7:9,097,206 bp) falling within a QTL for inflorescence width and the later SNP (Chr7:44,198,228 bp) associating with dry inflorescence weight (Zhang *et al.* 2015). It is possible that the identified SNPs are either novel sources of variation or are in LD with known inflorescence traits. Last, the putative identification of waxy in the multivariate GWAS may result from either differing starch concentrations or composition in the grain or the SNP may simply fall within a common linkage block. The alleles putatively associated with *waxy* segregated in PI22913 and PI586454 RIL families both of which are sweet sorghums with bicolor and kafir-bicolor racial backgrounds. It may be that as more carbon is allocated to starch production and concentrations increase, pericarp color may lighten since pure starch is white. The impacts of alterations in carbon partitioning may confer either large systemic changes—as seen between different sorghum types—or small changes—as even a trait as innocuous as perceived pericarp color could be subject to change.

We created a NAM population in sorghum that specifically captures the contrasting phenotypic traits needed to characterize, define, and model the complexities of carbon fixation, translocation, and utilization within an amendable model system. The dynamics of these carbon partitioning features can all be captured by this population because it incorporates every sorghum type in a structured population that can be used for traditional linkage or association mapping, eQTL studies, or physiological and agronomic modeling experiments (Guo *et al.* 2010; Slewinski 2012; Irving 2015). Each type is defined by its own source/sink relationship and compositional construction which influences plant metabolism, photosynthetic capability, and carbon fixation and sequestration potential (Irving 2015). As more emphasis is placed on the role of agronomy, management, and crop selection in carbon sequestration, a suitable model will be needed to fully elucidate the complex interactions that define the carbon costs and benefits of cropping options, especially as it relates to sorghum (Popp *et al.* 2011; Hammer *et al.* 2019).

In summary, the CP-NAM provides unique benefits to researchers and scientists seeking to understand, characterize, and exploit plant systems to increase overall productivity and tailor agronomic crops for specific usages that ultimately increase the availability of nutritious food and sustainable feedstocks to address both the shortage of arable land and the continuous release of greenhouse gas emissions from human activities. The creation and characterization of this population addresses the fundamental lack of genomic resources for nonfood usages of crop species and shifts focus from the entrenched emphasis on grain production to a more robust system tailored to overall productivity, which could ultimately lead to yield gains in food and feed production. The CP-NAM can serve as a fundamental resource to explicate the relationship among carbon fixation, sequestration, and productivity to create crops for both the traditional and future needs of agricultural production.
